# Enhancing skill development in the medical kitchen: an in-depth exploration through the experiences of undergraduate medical students

**DOI:** 10.1186/s12909-025-08123-5

**Published:** 2025-11-04

**Authors:** Suwimol Prusmetikul, Jakub L Radzikowski, C. Sofia Chacón González, Aynkaran Dharmarajah, Roger L Kneebone

**Affiliations:** 1 Orthopaedic Department, Faculty of Medicine, Ramathibodi Hospital, Mahidol University, Bangkok, Thailand; 2 Department of Chemistry, Faculty of Natural Sciences, Imperial College London, London, United Kingdom; 3 Department of Surgery and Cancer, Faculty of Medicine, Imperial College London, London, United Kingdom; 4 Faculty of Medicine, School of Medicine, Imperial College London, London, United Kingdom

**Keywords:** Skill development, Simulation, Transdisciplinary, Undergraduate, Medical kitchen

## Abstract

**Background:**

The Medical Kitchen course was developed to facilitate the transition of second-year medical students from theoretical learning to clinical placements by integrating practical skill training within a transdisciplinary approach. This study explored two research questions: how students perceived their technical and nontechnical skill development during the course, and which course features could be optimized to better support this development.

**Methods:**

Thirteen medical students who attended the course participated in three focus group discussions. Transcripts were analyzed using thematic analysis to identify themes and sub-themes related to skill development.

**Results:**

Six sub-themes emerged from the analysis, categorized under two main themes: technical and nontechnical skill development. Participants reported that the course significantly enhanced their technical skills, particularly in suturing, through hands-on activities and a supportive learning environment. They highlighted the importance of continuous practice to achieve proficiency. The group setting facilitated collaborative learning and recognition of diverse learning styles. Additionally, the course helped students recognize the significance of nontechnical skills, which they had previously overlooked, emphasizing their relevance for future clinical practice. While participants expressed a desire for more explicit instruction and structured opportunities to develop both technical and nontechnical skills, they recognized the value of these competencies and their impact on future professional development.

**Conclusion:**

From the students’ perspectives, the Medical Kitchen course provided valuable opportunities to develop technical skill while also enhancing awareness of the importance of nontechnical skills in clinical practice. Further course refinements could optimize skill acquisition by offering clearer explanation of the transdisciplinary approach and incorporating additional structured learning elements and practice opportunities, thereby better supporting students’ transition to clinical placements.

**Supplementary Information:**

The online version contains supplementary material available at 10.1186/s12909-025-08123-5.

## Background

Simulation is widely used in medical education to enhance students’ learning across various domains, including procedural skills, communication, or critical thinking [1]. Various simulation tools are purposefully designed and implemented to align with specific learning objectives. For example, simulated patients help students practice clinical communication, while silicone models replicate human skin for suturing practice. More advanced designs integrate these elements by placing silicone models on simulated patients, enabling students to develop both procedural and communication skills within realistic clinical scenarios.

The Medical Kitchen is a transdisciplinary simulation course designed for second-year medical students. Aligned with the curriculum of Faculty of Medicine at Imperial College London, the course is strategically positioned during the transition from preclinical studies in the second year to clinical exposure in the third year, taking place during the study break between these training phases. The Medical Kitchen integrates culinary and clinical skills practice, which comprises two main activities: vegetable turning and suturing on a silicone model [2]. The vegetable turning activity is deliberately selected as a simple and familiar culinary task, reflecting a skill many students may have encountered through basic cooking techniques or routine culinary experiences. This familiarity serves to shift the learning environment away from complex clinical settings, thereby reducing the cognitive load typically imposed by advanced clinical scenarios. By minimizing extraneous cognitive demands, the activity enables students to concentrate on refining fine motor skills and hand dexterity within a low-stakes environment [3].

Jean Piaget coined the term “transdisciplinarity” and clarified it alongside other epistemological terms, such as multidisciplinarity and interdisciplinarity [4]. Transdisciplinarity, distinct from multidisciplinarity and interdisciplinarity, eliminates disciplinary boundaries to create a unified approach to complex problems. It leverages shared skills and perspectives across fields, uncovering overlooked practices and enhancing quality and safety. Transdisciplinary enquiry not only complements but also surpasses multidisciplinary and interdisciplinary approaches by fostering holistic understanding and innovative solutions [4, 5].

For example, mise-en-place (a fundamental concept in culinary training that involves organizing a workspace and preparing instruments [6]) reflects the preparatory steps required in medical procedures. While mise-en-place is a cornerstone of chef training, its equivalent in medical education has been less emphasized. By integrating this principle, the course reinforces the importance of preparation, professionalism, mindfulness, and respect for materials and colleagues [6].

The course is structured to develop essential psychomotor and transferable skills crucial in clinical practice, including equipment preparation, adherence to safety protocols, hygiene techniques, and effective communication, in alignment with the standards set by the General Medical Council [7, 8]. Instead of utilizing conventional clinical simulations, this course adopts a transdisciplinary approach by incorporating culinary activities that parallel fundamental clinical procedures. Central to this design is the vegetable-turning task, which not only emulates clinical hand skills involved in suturing. To illustrate, the controlled wrist movements required during vegetable turning closely resemble the wrist actions necessary for suturing. Additionally, the varied textures of different vegetables, such as the smooth surface of courgettes contrasted with the firmness of raw potatoes, provide subtle tactile feedback analogous to the diverse skin textures found in patients across different age groups. By drawing these parallels, the course promotes the meaningful transfer of skills from a familiar, non-clinical context to clinical procedures, thereby enhancing student engagement and skill development.

The vegetable turning situates learning within a familiar and culturally embedded practice, thereby reducing cognitive load and supporting skill acquisition in a low-stakes environment. This cultivation of both technical proficiency and cultural responsiveness aligns with transdisciplinary education’s aim to integrate transferrable knowledge across domains, reinforcing student engagement and confidence [2]. Beyond the formal session, students are encouraged to further develop these skills using ordinary materials and tools available at home, an approach that proved applicable previously during period of remote learning, such as the COVID-19 pandemic [2]. The vegetable-turning task serves not only as a psychomotor scaffold but also as a means to nurture professional habits, situational preparedness, and reflective practice foundational to clinical excellence. By highlighting both the familiarity and the cultural situatedness of the task, the course supports learners in bridging non-clinical motor skills with clinical applications in a culturally responsive way.

Recent studies demonstrated the adoption of transdisciplinary education in health professions, characterized by the dissolution of traditional disciplinary boundaries and integration of knowledge, methods, and values. For instance, novel transdisciplinary dual degree curricula intentionally merged disciplines such as medicine and engineering, fostering cohesive learning environments that cultivate innovative problem-solving skills and professional identity formation [9]. Another example is training health students in improvisational skills inspired by improvisational theater. The program aimed to enhance skills in expressing emotions, decision-making, listening, and adapting to unpredictable events. The simulation integrated health, arts, and psychology, and students found it valuable for developing communication skills applicable in interactions with patients and their relatives [10]. Furthermore, a scoping review highlighted transdisciplinary education’s emphasis on reflexive collaboration and knowledge co-creation, facilitating holistic and context-sensitive learning necessary for complex healthcare challenges [11]. These developments support the Medical Kitchen’s transdisciplinary approach, which integrates culinary and clinical skills within a unified educational framework to enhance skill transfer and learner engagement.

Before the course, each student receives a manual outlining its aims, learning objectives, guidelines, task details, and QR codes linking to demonstration videos (Appendix 1). Throughout the course, students engage in individual and paired practice sessions [2]. In paired sessions, students take turns performing the role of doctor or patient. While performing the procedure, students should act as doctors and communicate with their peers appropriately, mimicking the situation of doctor-patient communication. These sessions are complemented by formative evaluations which were marked by their pairs, followed by reflective sessions led by facilitators to aid students in consolidating their learning points [12].

Fitts and Posner’s [13] three-stage model of skill acquisition, comprising the cognitive, associative, and autonomous stages, provides a conceptual foundation for the design of this course. In the cognitive stage, learners focus on understanding and following the sequence of a new skill. As they transition into the associative stage, their attention shifts to refining the quality of performance, which would become smoother and requiring less conscious effort. Finally, when achieving autonomous stage, learners can perform the skill efficiently with minimal conscious thought. The design of later session, where students perform the technical skill while simultaneously communicating with their pairs, introduces additional complexity, mirroring real-world clinical environments where multitasking is essential. To effectively manage both tasks simultaneously, individuals must attain a certain level of proficiency in the technical skill, allowing them to perform it with less cognitive demand. Although students in this course are not expected to reach the autonomous stage or have expertise in these skills, they are introduced to the iterative nature of skill development. This approach emphasizes the importance of ongoing practice, helping students appreciate how foundational skills can be refined and integrated with nontechnical skills throughout their professional growth.

More than focusing solely on technical or nontechnical skills, the course serves as an introduction to the transition from theoretical knowledge to hands-on clinical application. By employing a transdisciplinary framework, the module draws on principles from the culinary arts to cultivate key competencies such as manual dexterity, situation awareness, and effective communication- skills fundamental to clinical practice. Such cross-disciplinary approaches have demonstrated their potential to enrich medical learning by providing alternative frameworks for expertise development, fostering adaptability, and enhancing problem-solving abilities [14, 15].

Considering that pre-clinical students are novice learners with limited exposure to complex medical knowledge, the transition from theoretical learning to clinical placements presents a potential cognitive challenge. Managing cognitive load during this period is essential to ensure effective skill acquisition. The Medical Kitchen module is therefore purposefully designed with simple and familiar learning tools, a clear structure of activities, and minimal reliance on intricate medical concepts [14]. This approach enables students to concentrate on core learning objectives, developing essential procedural skills without the pressure of high-stakes clinical environments.

**Fig. 1:**
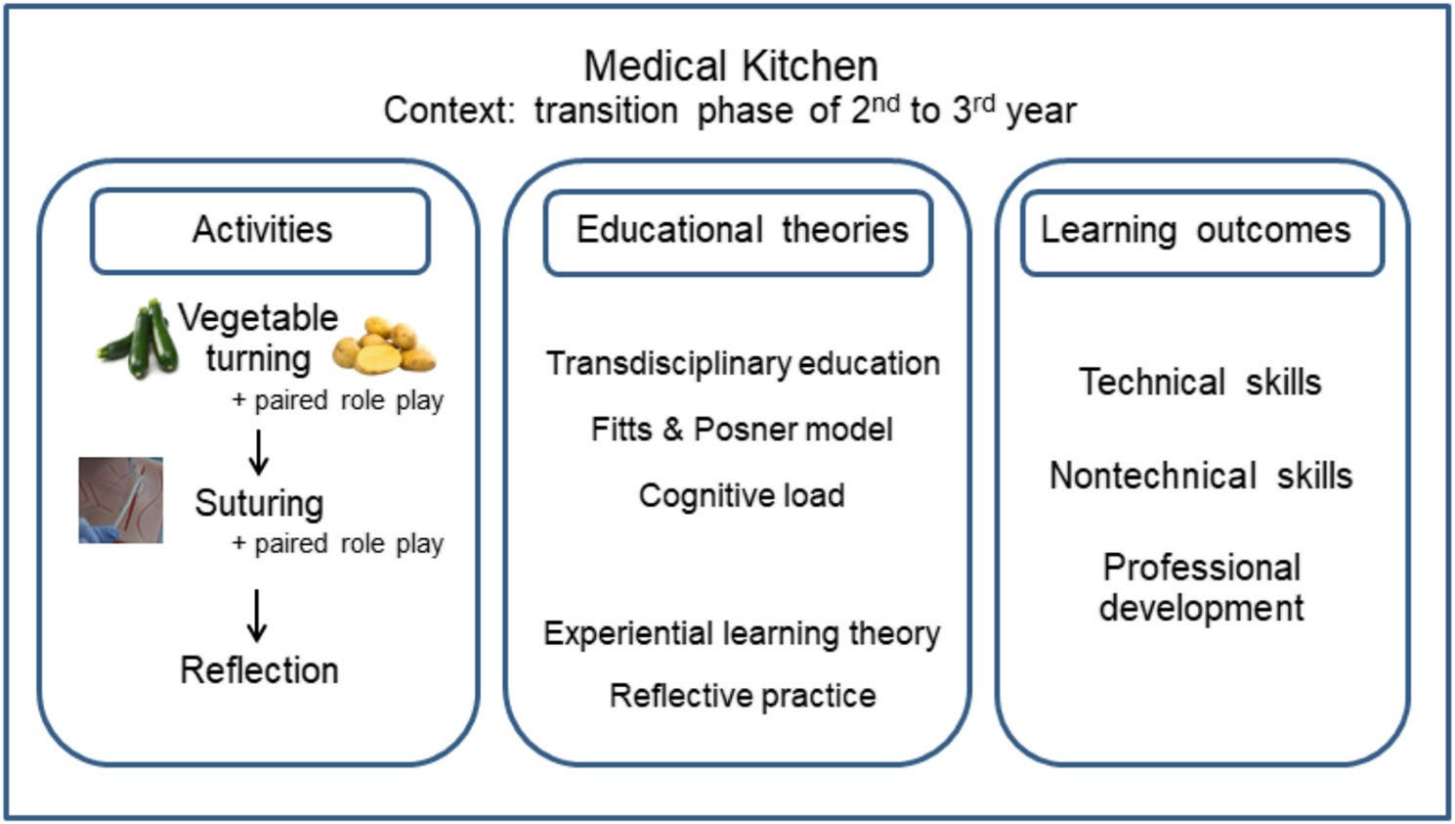
Conceptual diagram of the designed activities in medical kitchen course, foundational educational theories, and expected learning outcomes

Through structured, low-stakes experiential learning, students engage in culinary tasks that parallel medical procedures, facilitating the interplay between cognitive and practical skills in a supportive environment. This method not only reduces the anxiety often associated with clinical learning but also underscores the broader educational value of transdisciplinary approaches in medical training. In this way, the Medical Kitchen acts as a scaffolded introduction to clinical learning, allowing students to build confidence, refine their skills, and develop a deeper appreciation of the complexities of professional practice. This diagram summarizes the Medical Kitchen course components, linking key activities to foundational educational theories and targeted learning outcomes (Fig. 1).

Despite its innovative approach, a gap remains in understanding the course’s impact on student learning outcomes, particularly in the context of technical and nontechnical skill development. Previous evaluations have primarily highlighted the course’s overall effectiveness through written reflective forms [2], yet limited study has captured students’ authentic perspectives on their shared learning experiences through group discussions, especially regarding the perceived benefits for skill development. Furthermore, the extent to which the Medical Kitchen fosters nontechnical skills and prepares students for clinical placements has not been comprehensively explored.

To address this gap, the present study is guided by two research questions: [1] How do early undergraduate medical students perceive their acquisition of technical or nontechnical skills during transdisciplinary simulation in the Medical Kitchen? and [2] What features of the Medical Kitchen should be maintained or enhanced to optimize its effectiveness in terms of contributing to students’ skill development?

## Methods

### Study design

This qualitative study employed a phenomenological approach to explore students’ lived experiences within the Medical Kitchen course, aiming to capture their perspectives and reflections on learning outcomes. Phenomenology facilitates a rich and in-depth understanding of how learners perceive, interpret, and find meanings in their engagement with the course activities [16]. We engaged in ongoing bracketing to set aside preconceived assumptions and conducted iterative reflection on the data to reveal patterns and meanings articulated by participants.

Since the data centered on participants’ reflections and insights, it was analyzed through a constructivist lens. Within the constructivist paradigm, reality is understood as a mental construct formed by individuals based on their prior experiences, foundational knowledge, and social interactions [17]. Consequently, the knowledge derived from personal experiences does not necessarily correspond with an external objective reality, but rather reflects how individuals make sense of their world. By adopting this paradigm, this study endeavors to delve deeply into participants’ perspectives, aiming to gain a nuanced understanding of their learning processes that align with the core principles of constructivism.

### Participant recruitment

Participants were volunteers from approximately 360 students who completed the Medical Kitchen course, recruited via posters, newsletters, and verbal announcements communicated to the entire eligible cohort. Thirteen students participated in three focus groups of 4–5 participants each. All participants received a participant information sheet and a list of questions (Appendix 2) via email prior to the meeting. The study’s objectives and details were verbally reiterated on the meeting day to ensure participants’ informed consent.

### Data collection method

This study used focus group discussions, enabling participants to share and build on each other’s perspectives. A semi-structured question format in an informal setting fostered open and candid dialogue. The main questions, provided to participants in advance, were carefully designed to align with the research objectives. (Appendix 2) Additionally, follow-up questions were included as needed to elicit further clarification or confirmation. The primary researcher (SP) served as the facilitator for all focus group sessions.

### Data analysis

Focus group recordings were transcribed verbatim and analyzed using QSR NVivo V.14.23.3, following the thematic analysis method outlined by Braun and Clarke [18]. Coding was guided by phenomenological reduction, focusing on capturing the core essence and meaning of participants’ lived experiences. Themes were developed to reflect how participants made sense of their learning within the phenomenological framework.

Initial coding was conducted by the primary researcher (SP), concentrating on concrete meanings within the transcripts. Early coding yielded numerous scattered codes that were challenging to synthesize into coherent themes. Through multiple rounds of re-coding and collaborative refinement with the co-researcher (RK), codes and themes were iteratively refined to ensure alignment with the research objectives and questions. The co-researcher (RK) reviewed initial codes and themes, and any disagreements were resolved through discussion, enhancing the credibility and rigour of the final thematic structure. Consistent with reflexive thematic analysis, intercoder agreement statistics were not performed, as coding is viewed as a fluid, interpretative process. Data sufficiency was identified through iterative coding until no new themes emerged, indicating saturation.

### Reflexivity statement

As both focus group facilitator and analyst, the primary researcher acknowledged the potential for bias influencing data collection and interpretation. Guided by the phenomenological principles, reflexivity was maintained through a reflective journal documenting assumptions, decisions, and emotional responses. Bracketing was integral to minimize preconceptions and promote attentiveness to participants’ authentic meanings and experiences. Collaborative analysis with co-researchers provided critical oversight, balancing the researcher’s clinical and educational perspectives, which were recognized as both resource and potentially bias. Transparent disclosure of this positionality strengthened the study’s credibility and trustworthiness of the findings.

## Results

Thirteen medical students who had previously attended the Medical Kitchen course participated in this study: eight females (61.5%) and five males (38.5%), aged 19–23 years. They were organized into 3 focus groups based on schedule availability. Participant demographic data are summarized in Table 1.

**Table 1:** Participant demographic data

Group	Year of Study	*N*	Female	Male	Age Range
A	Beginning of Year 3	4	2	2	20–22
B	Beginning of Year 3	5	3	2	19–23
C	Completed Year 3 (within 1 month)	4	3	1	20–22
Total	-	13	8	5	19–23

These participants represented all responses received from voluntary recruitment efforts through posters, newsletters, and announcements. Recruitment and data collection were conducted between May and July 2024 (3 months), as permitted by ethical approval. This timeframe constrained the number of volunteers available for enrollment.

Twenty-four descriptive open codes were generated from the transcripts using thematic analysis. These codes were synthesized into six sub-themes, which were further categorized under two major themes corresponding to the primary research question: technical and nontechnical skill development. A summary of these themes is presented in Table 2.

**Table 2:** Themes and sub-themes of data

Themes	Sub-themes
Technical Skill Development	Deliberate Practice with Structured Supervision
	Personal Learning Styles and Methods
	Supportive Learning Environment with Peer Collaboration
	Facilitating Skill Transition from Novice to Clinical Placement
Nontechnical Skill Development	Recognizing the Necessity of Nontechnical Skills for Future Clinical Practice
	Fostering the Development of Professional Attributes in Clinical Practice

### Theme one: technical skill development

#### Deliberate practice with structured supervision

Participants from groups A and B recognized the difficulties of performing new skills, particularly as many had previously only been exposed to theoretical study. Despite the challenges, they acknowledged the opportunity for skill development and learning progression.

Participants from all groups valued the course’s hands-on approach, emphasizing the effectiveness of detailed instruction and structured guidance. They particularly appreciated the consultants’ expertise and advice, which they recognized as essential for skill development.

“It felt very hands on. We were being taught exactly how to do it. So it’s the first time we’ve been taught to that kind of level of guidance and there were people coming around, so I think it’s really good.” (B3).

“It was good to hear like how to do suturing from a consultant surgeon, even like the basics of how to hold the needle holder and how to perform simple interrupted sutures.” (A4).

They recognized the importance of ongoing, repeated practice for developing their technical skills by performing the skills themselves and observing how consultants executed the same skill.

“Obviously, watching the doctor does it fantastically, but you have to remember that people aren’t born like that. (Consultant) done a lot of practice. Therefore, if you want to get good at anything but this, probably have to practice, a fair amount regularly over a period of time.” (B5).

Participants valued the opportunity for repeated hands-on practice, viewing it as a crucial component of skill acquisition. They recognized the importance of learning from their mistakes, gaining valuable insights to refine their techniques for future practice.

“There is no other real opportunity to learn how to suture. So I think it’s very well structured for that and I think you do leave the session knowing how to confidently enough throw as many as you need to.” (C1).

“You will make a mistake it’s probably the best time to make a mistake. That’s perfectly normal, but actually it doesn’t mean anything is wrong. But it was a good place to start learning from.” (B5).

However, group A participants felt practice time was too short and suggested extending it by reducing time from other sections. Group B participants mentioned a lack of clear instructions on how to structure their practice time, especially during role-play section, which they felt hindered their learning.

“I was suturing, suturing, suturing and I was listening to the patient saying they’re in pain. I was reassuring them. (…) And then suddenly it was like, OK, in 5 minutes we’re gonna do reflection so we had to swap over… after 25 minutes. But if they said like, we’ve got 20 minutes with this task. First 10 minutes is for one person, 2nd 10 minutes is the other person then you feel like… at least you make sure both of us get equal time.” (B3).

Most participants in groups A and B preferred active engagement from consultants, who would ask if they needed help or stop by to observe and demonstrate skills. Participants pointed out that students might not recognize their errors or mistakes, so it would be beneficial if consultants observed and provided feedback. Additionally, some students were reluctant to ask for help to avoid standing out from the group.

Participants developed insights into the need for continuous practice to prepare for upcoming clinical placements. They expressed a desire for consultant advice to assist in their continuous practice, particularly during breaks between study years. They expressed a desire for structured guidance and consultant advice on the frequency of practicing clinical skills, along with access to specific resources or practice kits. While some were willing to invest in their own practice materials, they highlighted the need for clear recommendations and targeted resources, beyond general online videos, such as guidance on different suturing techniques to support ongoing skill development.

#### Personal learning styles and methods

Participants from groups A and B noted their preferences or styles of learning from the course structure. One participant recognized that the various teaching strategies in the course could facilitate students’ learning in different ways.

“It teaches you a lot about how you learn as well and like the environment that you’re in, so for example who like… if you decided to watch the person doing it and then you follow that, or if you would prefer to have someone at your table while you were doing it like, I think it was really good cause you had both of those options.” (B3).

Participants also recognized the usefulness of learning materials varied for each individual. They proposed different strategies for using existing learning materials and additional learning materials to enhance their learning quality.

“People find people do things differently, like, some of them really find it useful to hear the instructions, but… that just I can’t understand into that at all.” (B2).

For example, participant A1 thought the pre-course video might not be helpful for them. Still, participant A2 noted its usefulness in preparing themselves and assisting in course structure by playing the video along while performing hands-on practice. Another example from participants was providing written manual regarding the details of procedural skill to tag the important points for them.

“We do know you have to be quite specific when it comes to explaining a procedure and so when it comes to, let’s say… simple interrupted sutures for wound and what exactly do you have to explain or like a structure maybe to follow would have been good.” (A4).

#### Supportive learning environment with peer collaboration

Participants from all groups noted that the course provided a low-pressure, safe, and fun environment, which they found enjoyable and supportive of their skill acquisition.

“Maintain that relaxing environment so you know if you do make mistakes, it’s not like hospital setting where you’re under pressure.” (B4).

They mentioned the benefits of a group setting as a factor supporting a safe learning environment within the course.

“I think it’s good doing it in the groups cause it also makes it more fun, and then everyone can kind of like help each other out or give each other tips which is quite useful by that it was good.” (C3).

Participants could learn and exchange their knowledge and techniques during the sessions through this group setting. Additionally, they were able to correct each other’s mistakes and used this collaborative learning method as another strategy to enhance their skill acquisition.

“I think as everyone has different levels of experience. So in our groups some people used to do it before some of us had never tried it, so I think it was really helpful just to ask each other questions as well.” (A3).

“I think it was quite good with the group based where it was hard to learn skill, but the fact that appears around you just helped correct each other mistakes.” (B4).

#### Facilitating skill transition from novice to clinical placement

All participants from group C acknowledged the course’s significant role in preparing them for clinical placements in their third year. From their experiences, they emphasized that this course is the only one specifically designed to cover technical skills for all students at an appropriate stage in their studies, unlike other third-year sessions which were often either poorly timed or focused more on assessment than hands-on practice or skill mastery.

“When you’re on placement, you have a clinical skills session every week that they would go through such suturing, but depending on the timetable, you might have already had your surgical placement. So everyone’s on in second year. That’s the only time. And then at some point during your third year, you get more specialized teaching, but you don’t know when that would be.” (C2).

“I think the clinical skill teaching of suturing in 3rd year is much less about the actual skill and a lot more about the script per se. You need to learn to get all the marks in your OSCE. It’s very much focused the exam and not to the actual skill in practice. So I think the actual medical kitchen aspect of it was far more useful for learning to suture than the clinical skill session.” (C1).

Participants from every group recognized the benefits of practicing suturing skills, knowing they would need to perform the skills during third-year studies. Apart from suturing, participants also noted other valuable learning points, such as workspace preparation and performing procedures on different materials that mimic texture of human skin. They suggested expanding the course to include more clinical techniques such as injections or surgical etiquette like sterile technique.

Although the vegetable turning task was intentionally selected as a basic and familiar task to reduce cognitive load for novice learners and develop fine motor skills, several participants expressed uncertainty about its connection to clinical procedures like suturing. The rationale behind this activity was not clearly communicated, which led to confusion and varying interpretations of its purpose.

“The connection between the two sessions wasn’t immediately obvious, and the explanation for the first session as in like why we were actually doing it wasn’t obvious, like it wasn’t clear.” (B1).

“It was nice to do something practical, but I think if you’re gonna keep the vegetable peeling thing, then I think there needs to be a better explanation of like the relevance, because I think a lot of us were kind of just a bit confused.” (C4).

In the absence of a clear explanation, participants generated their own assumptions to make sense of the activity, such as relating it to practicing scalpel technique due to the fine wrist movement and instruments involved.

“I think the only thing I could kind of relate it to, was the scalpel technique of like using quite slippery small technique of your hand and fine movements. But I don’t know if that could be done in a different way.” (C2).

These reflections suggest that clearer articulation of learning objectives and relevance could enhance student engagement and support meaningful transfer of skills.

### Theme two: nontechnical skill development

#### Recognizing the necessity of nontechnical skills for future clinical practice

Participants from groups A and B, who had spent the first two years immersed in theoretical studies, acknowledged that the course provided valuable insights into the practical aspects of medical practice. They recognized that being a practitioner involves more than just performing physical tasks or possessing academic knowledge; it requires integrating nontechnical skills into their practice.

“I thought that was quite useful as well because it reminds you that… suturing is not just something you do by itself like it… How it fits into the bigger picture. So I thought that was quite useful because I definitely found it harder to suture the second time round when I had to interact with the patient as well.” (A3).

“I think it was nice to revisit the fact that medicine isn’t just regular learning or just figure out, especially after exams. (___) I think that was nice to get back to bring that to surface because first two years non-clinical you’re learning a lot and it might feel like this isn’t what you signed up for, but getting back to some other aspects of medicine, more handling… feel like that was… it was important and it was probably necessary at the point that we are.” (B5).

Role-playing as patients further enriched their understanding by allowing them to reflect not only from the practitioner’s viewpoint but also from the patient’s experience during procedures. These activities emphasized the cognitive and emotional demands placed on practitioners when simultaneously managing technical tasks and patient interaction.

“When you’re like… sewing someone up, which is obviously I think, more distressing for the patient.” (B2).

Collectively, these reflections suggested a growing awareness among students that technical and nontechnical skills are interdependent. Some participants began to realize that mastering technical skills is an essential foundation to effectively incorporate nontechnical skills into their clinical practice.

“The second part was really interesting when you have like, a patient because I found that as soon as somebody started talking to me, I was like… I couldn’t do it properly after like… some distracting me.” (B3).

“When we were doing the role play… for both the vegetable turning and the suturing, it was really striking to me how well you have to know the procedure itself to be able to talk with the patients throughout the time you’re performing it. Otherwise we felt with (another student), who was my partner in the role play. If you were not talking at all times and you were just focusing on the procedure itself, it would feel very like extremely awkward because it would be like long silent process” (A1).

#### Fostering the development of professional attributes in clinical practice

Participants from groups A and B expressed a desire for more in-depth discussions on the concept of professional attributes that define a good practitioner. While the professional attributes were mentioned in many other modules, participants felt these discussions were often superficial and repetitive, lacking depth or practical application.

“Cause we’ve been asked that… a lot. So I think sometimes you get to…if you don’t explore it further, you just kind of just listing it. Yeah. We know that’s the answer.” (B3).

“Building on that like for the second session, when we had to like… give one word to describe what made good doctor for this, again it became like a vocabulary exercise again, because like… we could have repeated the same word.” (A4).

Participants indicated a need for more targeted and contextualized discussions about professional attributes. They wanted clearer guidance on which characteristics matter most, how these attributes might be required in specific circumstances, and practical steps for cultivating these professional qualities.

“Let’s say we’ve got 3 adjectives. Why do we need them to have this? How are you to make sure that you have this? What would you do if you didn’t have this?” (B3).

“Maybe also like in different situations. (…) So maybe I would have to deliver news, do a surgery. You want doctor to be these three qualities or maybe in non-surgery one. You want your doctor to be having these three best qualities.” (B4).

One participant (A3) suggested that discussing the preferred attributes of doctors before engaging in role-play could have made the role-play more effective, allowing them to apply these qualities during the exercise consciously.

## Discussion

This study investigated how medical students perceive the Medical Kitchen course’s effectiveness in fostering technical and nontechnical skills. The following discussion will delve into each identified sub-theme, drawing connections between the participants’ insights and relevant educational theories and literature to understand the course’s impact comprehensively. Additionally, the researchers highlighted key implications for future simulation development to broaden educators’ perspectives in designing practical learning activities.

### Theme one: technical skill development

#### Deliberate practice with structured supervision

Participants across all groups valued the course’s hands-on, repeated practice with close supervision from consultants. Groups A and B, who had more recent course experiences and were approaching clinical placements, expressed the challenges of acquiring new skills and recognized the importance of continuous, intentional practice in preparing for upcoming clinical responsibilities. These insights aligned with the deliberate practice concept proposed by Ericsson [19], which highlights purposeful, well-designed tasks, immediate feedback, and repeated practice to foster technical expertise.

The Medical Kitchen course implemented these elements through structured specific tasks, real-time educator feedback, and refining skills through repetition. This iterative process enabled students to progressively refine their techniques and develop confidence. Participants also acknowledged the necessity of extending deliberate practice beyond the course to maintain and enhance clinical skills.

Participants suggested improvements to better support learning. First, they felt the hands-on practice time was too short and that unclear session instructions led to unequal practice opportunities. Second, they preferred more proactive consultant engagement, as passive observation might limit error recognition and timely assistance. Finally, they requested guidance on continued skill development beyond the course, such as access to learning resources and procedural kits.

*Implication* - Educator Development and Guidance:

Effective facilitation requires training educators to provide structured supervision, deliver timely feedback, and offer personalized support, thereby fostering continuous skill development and mastery.

#### Personal learning styles and methods

Participants from groups A and B expressed diverse experiences regarding the methods used to deliver course content and instructions. While some participants found pre-course videos helpful for preparation and reducing the need for in-class demonstrations, others perceived little benefit. Similarly, variation existed in how students understood consultant explanations, reflecting individual differences in processing information. These findings aligned with the concept of learning styles, which suggests that learners retain information and develop skills through preferred sensory modalities such as kinesthetic, auditory, visual, or reading/writing approaches, with some students favouring unimodal learning while others preferred multimodal methods [20–24].

Although the learning styles concept had been widely debated and lacks consensus on optimal instructional strategies [25], studies showed that medical students’ preferences may shift over time, as evaluated by Kolb’s Learning Style Inventory [26]. Variability in learning preferences might also arise due to contextual and prior learning experiences, emphasizing that learning styles are not fixed traits but dynamic and evolving. From a neuroscience perspective, learning styles reflected underlying neural processing pathways involving sensory registers, working memory, and long-term memory [27]. Neuroplasticity facilitated the strengthening of neural connections through repeated, multisensory engagement. Additionally, metacognitive skills enabled learners to monitor and adapt their strategies to optimize learning, further support the value of diverse and dynamic instructional methods [28].

Participants valued the course’s multiple instructional strategies and recommended supplementing procedural demonstrations with written instructions. Considering the challenges novice learners encountered in acquiring new skills, particularly in retaining procedural steps after a single demonstration, supplementary resources could provide valuable reinforcement and support skill retention.

*Implication* - Diverse Learning Resources:

To enhance comprehension and retention, instructional design should incorporate varied learning materials, including videos, written guides, and hands-on practice opportunities, catering to a spectrum of learning preferences and supporting the evolving nature of learners’ styles.

#### Supportive learning environment with peer collaboration

Participants from every group valued the safe, group-based learning environment that fostered peer-supported learning. This approach resonated with Vygotsky’s social constructivism, which posits that knowledge is constructed through social interactions and linguistic processes [29]. In such environments, learners collaboratively exchanged knowledge, skills, values, and attitudes, making learning more dynamic and enriching. This collaborative setting promoted essential skills such as communication, self-assessment, and peer assessment [30].

Participants described performing skills with peers as enjoyable and low-pressure. The interplay between a safe environment and peer learning enhanced learner engagement. Educators played a critical role as facilitators by monitoring sessions, encouraging interactions, and signaling availability to support learners, thereby maximizing effectiveness [30]. While well-structured activities with clear objectives are necessary to guide learning, flexibility was crucial to preserving a supportive, low-pressure atmosphere that encourages self-directed inquiry [30]. Striking this balance required educators to support intended learning outcomes while allowing deviation that fosters experiential learning. Navigating these complexities required a deep understanding the concept of peer-supported learning and may benefit from targeted facilitation training. As such, peer support functioned as a distinct yet complementary mechanism that enhances the quality of deliberate practice by creating a collaborative, motivational learning culture.

A recent systematic review of peer learning in healthcare education reinforced that peer-supported learning promotes deeper reflection, discussion, and autonomous learning [31]. However, implementation challenges included managing different learning styles, varying learner capabilities, and knowledge gaps, particularly among novice learners who may have limited prior experience for meaningful discussion. The Peer Instruction (PI) method, conceptualized by Eric Mazur, demonstrated clear benefits in fields of science, technology, engineering, and mathematics (STEM) by increasing engagement, critical thinking, and problem-solving. While PI remained less explored in medicine, possibly due to traditional preferences for didactic lectures or case-based learning, its potential to foster active, lifelong learning made it a promising model for medical education [32].

*Implication* - Promotion of Peer-Supported Learning:

To strengthen peer-supported learning, environments should be fostered that encourage peer interaction, constructive feedback, and collaborative problem-solving, thereby enhancing communication, teamwork, and critical thinking skills. Educators must be trained to facilitate this process effectively, balancing structure with learner autonomy and creating psychologically safe spaces for meaningful peer engagement.

#### Facilitating skill transition from novice to clinical placement

Participants across all groups found the course valuable for providing their first meaningful hands-on experience with suturing, a skill they recognized as essential for their third-year study. This understanding was confirmed by group C participants, who noted that practicing suturing in a focused, low-stakes environment allowed concentration on deliberate procedural mastery without the pressure of clinical scenarios or formal assessments. This approach effectively bridged the gap between pre-clinical theory learning and clinical demands, contrasting with more scripted, exam-oriented clinical skills sessions delivered in the third year.

The course’s transdisciplinary approach, integrating culinary tasks such as vegetable turning to develop fine motor control and cognitive load management, caused some initial confusion regarding its clinical relevance. Some students interpreted it narrowly as an introduction to scalpel handling, overlooking the broader aim of fostering procedural fluency. Given that this transdisciplinary method is innovative and unconventional in medical education, explicit articulation of its clinical relevance was crucial to avoid student disengagement or undervaluing the task [33].

The role-play component encouraged reflection on the cognitive and motor demands of multitasking during procedures, aligning with Fitts and Posner’s [13] three-stage model of skill acquisition, which describes the progression from cognitive to autonomous skill performance. The course introduced dual-task challenges and reinforced that technical competence is foundational to multitasking effectively in clinical settings.

To enhance clarity and engagement, educators should offer a clear explanation linking the vegetable-turning task to the procedural demands of suturing, for example, by emphasizing the shared wrist movements involved in both tasks. Such clarification would augment understanding of skill transferability and improve effectiveness of skill practice.

Participants also expressed desire for additional clinical skills training, such as alternative suturing techniques and injections, reflecting a need for supplementary modules to better support the transition to clinical placements.

*Implication* - Innovative Transdisciplinary Design:

Clear articulation of learning goals and clinical relevance of transdisciplinary activities are crucial to maintain student focus on key competencies. This explicit linkage fosters engagement, enhances skill transfer, and strengthens novice learners’ readiness for clinical practice.

### Theme two: nontechnical skill development

#### Recognizing the necessity of nontechnical skills for future clinical practice

Participants from groups A and B noted that the course broadened their understanding of clinical practice beyond theoretical knowledge. Role-playing as both doctor and patient highlighted the crucial role of nontechnical skills alongside technical proficiency. Participants’ reflections were analyzed inductively, and their recognition of the nontechnical skills’ value aligned with Mezirow’s [34] concept of transformative learning, whereby new experiences acted as ‘disorienting dilemmas’ prompting learners to critically reassess prior assumptions and adopt new perspectives on clinical challenges [35].

Until this experience, participants’ understanding was largely limited to theoretical studies. Role-play fostered early phases of transformative learning, such as ‘self-examination’ and ‘critical assessment of assumptions’, emphasizing nontechnical competence including communication, situation awareness, and emotional awareness [35]. Educators could support progression along this trajectory by creating safe, non-hierarchical environments conducive to authentic dialogue, discussion, and sharing of clinical experiences [36].

The role-play also nurtured patient-centeredness and emotional competence, reinforcing that technical skill mastery supports effective nontechnical performance. This view was consistent with Fitts and Posner’s [13] skill acquisition model, which situates technical competence as foundational for multitasking and cognitive demands in clinical settings.

Contemporary literature emphasized that nontechnical skills such as communication, decision-making, leadership, teamwork, and stress management are fundamental to safe and effective clinical care, complementing technical expertise and reducing medical errors [37, 38]. Early integration of nontechnical skills in curricula could strengthen competence, improve patient safety, and prepare learners for complexities of real-world practice.

To maximize the course’s impact, clearer teaching strategies linking technical skills with nontechnical competencies should be employed, helping students appreciate their interdependence in clinical practice.

*Implication* - Early Introduction of Clinical Learning:

Educators should embed clinical learning early in the curriculum, emphasizing development of nontechnical skills alongside technical skills, to better prepare students for the realities and demands of clinical placements.

#### Fostering the development of professional attributes in clinical practice

Participants from groups A and B, having recently completed the course, acknowledged the importance of its focus on professional attributes but found the sessions somewhat repetitive, as similar discussions had occurred in prior modules. They expressed a need for clearer guidance on how to cultivate the professional qualities discussed in the session and understanding which attributes are most critical in specific clinical circumstances. As they had not yet begun clinical placements, their understanding remained largely theoretical, and the repetitive format risked disengagement, underscoring the challenge of teaching professional identity formation in pre-clinical curricula.

While professional attributes were fundamental to medical training, no universal consensus existed on their definition, teaching methods, or assessment. O’Sullivan et al. [39] suggested that institutions should establish their own definitions to guide curriculum development, as professional values and expectations vary by culture and context [40]. Early introduction to core professional values could scaffold learners’ development and provide foundational frameworks for observing and internalizing essential professional attributes during clinical placements to prepare them for real-world practice.

A systematic review by Birden et al. [41] identified role modeling as the most effective method for teaching professionalism, with the concept grounded in situated learning theory. Role modeling influenced students through clinical and non-clinical experiences, shaping professional attitudes and values crucial for future practice [40]. Situated learning occurred as learners actively participate in social practices, gradually integrating into clinical teams or Community of Practice (CoP), beginning with peripheral roles and progressing toward full participation, a process termed Legitimate Peripheral Participation by Lave and Wenger [42]. Given that role modeling and situated learning predominantly took place during clinical experiences, their effectiveness was limited in preclinical years.

Although extensive literature explored methods to integrate professional attributes into medical curricula, analytical comparisons of different teaching strategies remained scarce. This lack of definitive evidence complicated identification of the most effective approaches and understanding of their theoretical underpinnings. Variations in professional attribute definitions and cultural contexts further hindered comparisons. Consequently, ongoing research is required to refine teaching methods that effectively support development of professional attributes.

*Implication* - Strategic Curriculum Integration:

Institutions should align learning objectives, teaching methods, and content cohesively across the curricula to promote clarity, minimize redundancy, and foster active student engagement. This strategic integration facilitates the development of professional attributes, enhances identity formation, and supports the transitions from theoretical learning to authentic clinical practice.

### Strengths and limitations

This study had several limitations. A primary limitation was the low participation rate relative to the eligible cohort, which inherently limited the transferability of findings. Recruitment through voluntary responses to posters, newsletters, and announcements, may introduce self-selection bias, as students with particular interests or availability were more likely to participate. Consequently, the participant sample may not fully represented the broader population and generalizability to other cohorts or contexts should be approached with caution.

The small sample size was also constrained by recruitment challenges and a limited research timeline. Nevertheless, the data captured a broad range of insights, providing valuable input for refining the Medical Kitchen course during this critical transition period.

Importantly, the focus group facilitator also contributed to data analysis, introducing potential bias. This influence was explicitly acknowledged and addressed through methodological reflexivity and bracketing, consistent with qualitative research rigor. The dual role of facilitator and analyst was recognized as a limitation but was managed through ongoing critical reflection and team discussions.

Another limitation was the absence of longitudinal follow-up to assess the course’s effectiveness across various stages of medical training. Our findings primarily reflected the course’s short-term impact, particularly on students’ skill development and readiness for initial clinical placements. The lack of follow-up had limited commentary on sustained or late-emerging outcomes, indicating a necessary avenue for future research.

Variation in focus group timing introduced potential recall bias. Participants interviewed shortly after course completion recalled specific activities and immediate learning outcomes, whereas those interviewed later reflected broader, experience-informed insights. While this temporal diversity enriched data depth and supported comprehensive analysis, it may affect response accuracy and consistency. We considered our sampling strategy a notable strength, which providing a spectrum of perspectives but acknowledged the trade-off in recall reliability.

As a preliminary exploration, these findings should be interpreted cautiously. Future research with larger, more diverse samples, and proactive recruitment was needed to improve representativeness and strengthen transferability. Subsequent studies might include expanded technical or culinary activities, integration with simulation training, and perspectives from faculty or clinical educators to comprehensively evaluate educational impact and clinical performance translation.

This study provided initial insights into the role of transdisciplinary education in supporting medical students’ skill development. Larger quantitative studies were warranted to evaluate the course’s educational impact further. Expanding course content and broadening outcome measures may enhance understanding of its effectiveness.

## Conclusion

From the students’ perspectives, the Medical Kitchen course offered valuable opportunities to practice technical skills and introduced effective strategies for continued skill enhancement beyond the course. Participants also acknowledged the significance of nontechnical skills for their future clinical practice and intended to develop these attributes further. Additional guidance and resources from educators were needed to support students in achieving their learning goals. While some students grasped the course’s key learning points, a more precise explanation of its transdisciplinary design could better align their focus with the intended learning objectives, enhancing overall effectiveness.

## Supplementary Information

Additional File 1: Appendix includes the Medical Kitchen course manual and list of questions for focus group discussion.

## Data Availability

The data that support the findings of this study are available from the corresponding author, Suwimol Prusmetikul, but restrictions apply to the availability of these data, which were used under license for the current study, and so are not publicly available. Data are however available from the authors upon reasonable request and access to the data is subject to the approval of the Imperial College Education Ethics Review Process (EERP) committee.
