# Supplementary material for: Enhancing skill development in the medical kitchen: an in-depth exploration through the experiences of undergraduate medical students
Source: BMC Med Educ. 2025 Nov 4;25:1541. doi: 10.1186/s12909-025-08123-5 (PMC12584502; doi:10.1186/s12909-025-08123-5)
Supplement: Supplementary file 1 — Additional File 1: Appendix includes the Medical Kitchen course manual and list of questions for focus group discussion. [file 12909_2025_8123_MOESM1_ESM.docx]

**Additional File: Appendix**

**
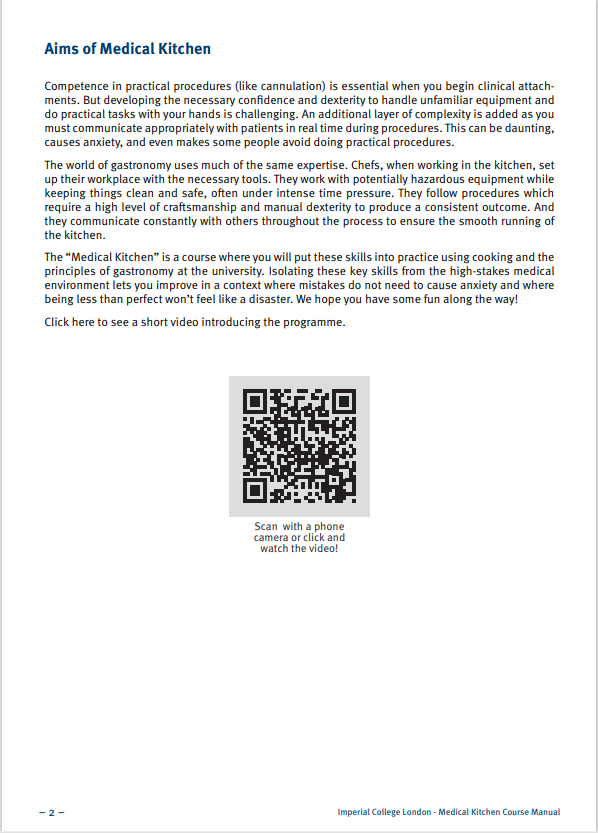

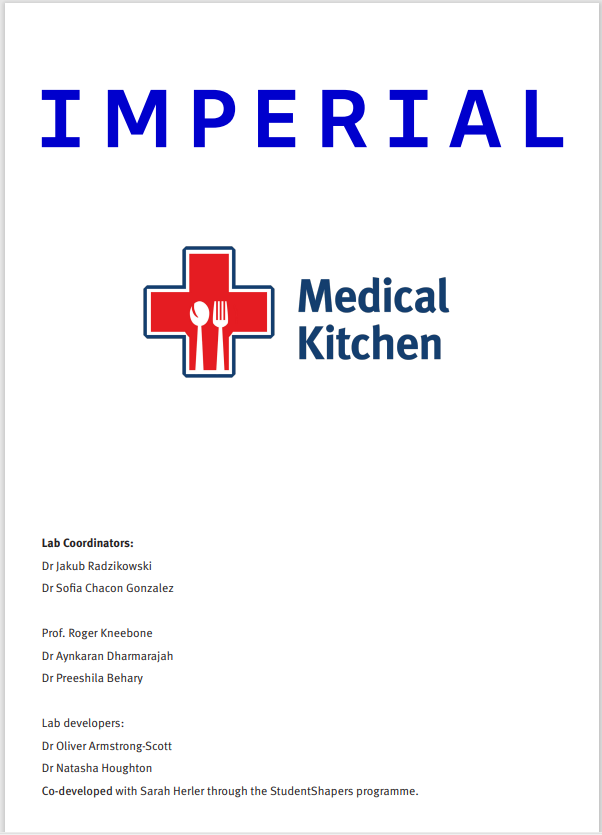
Appendix 1: The Medical Kitchen Course Manual**


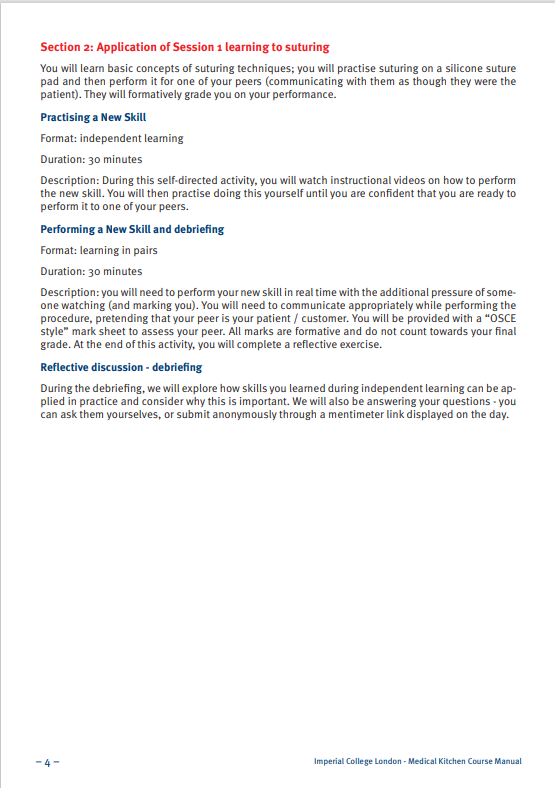

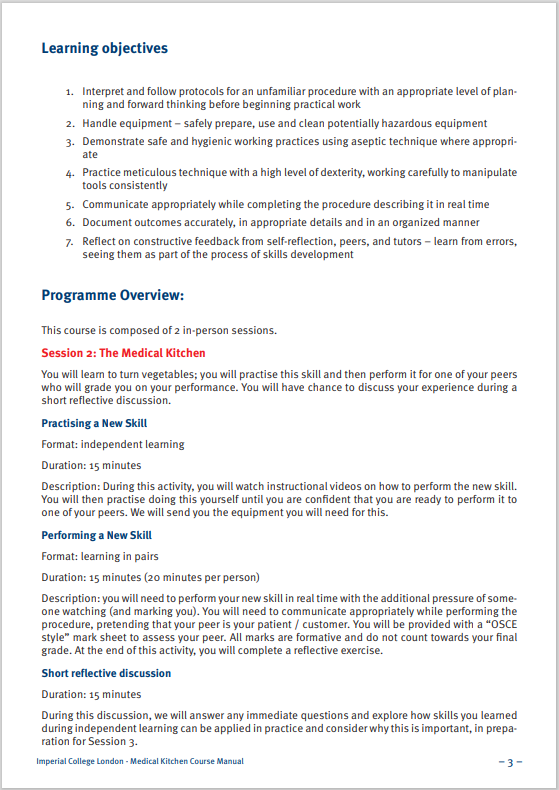


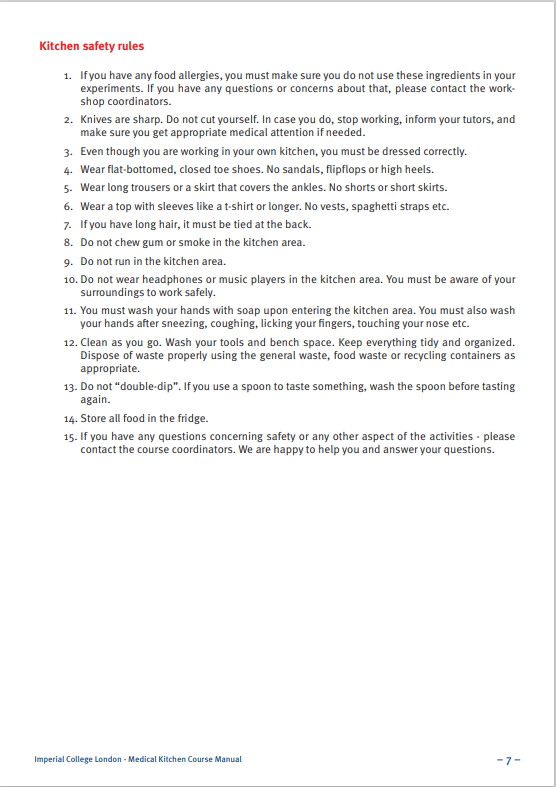

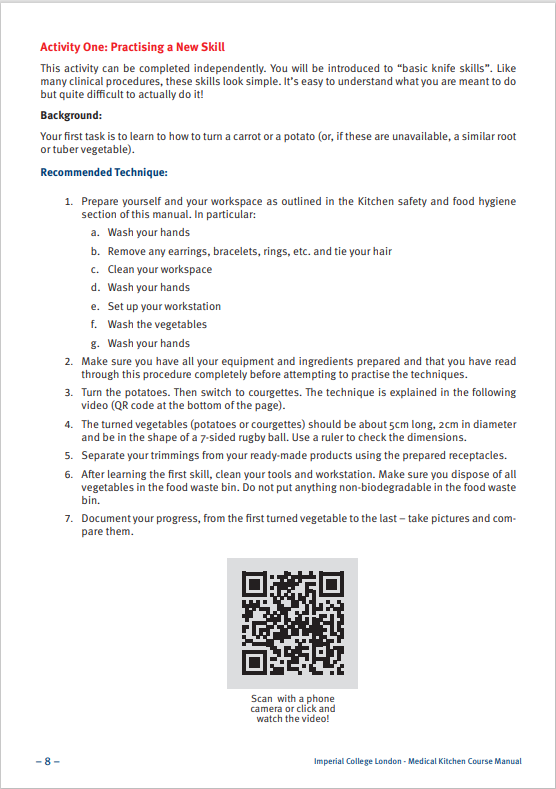

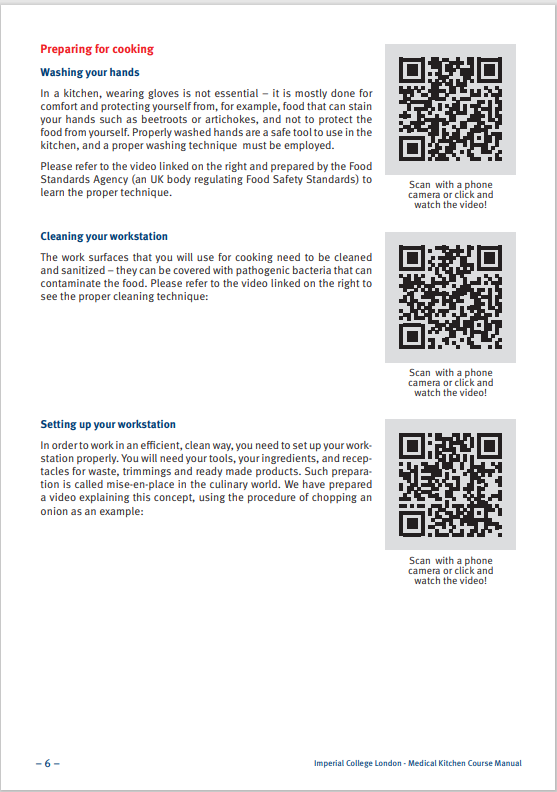

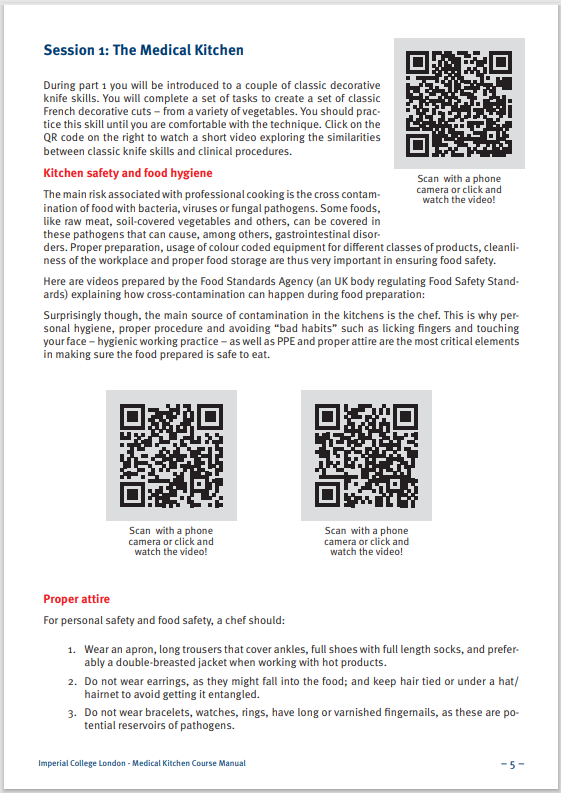


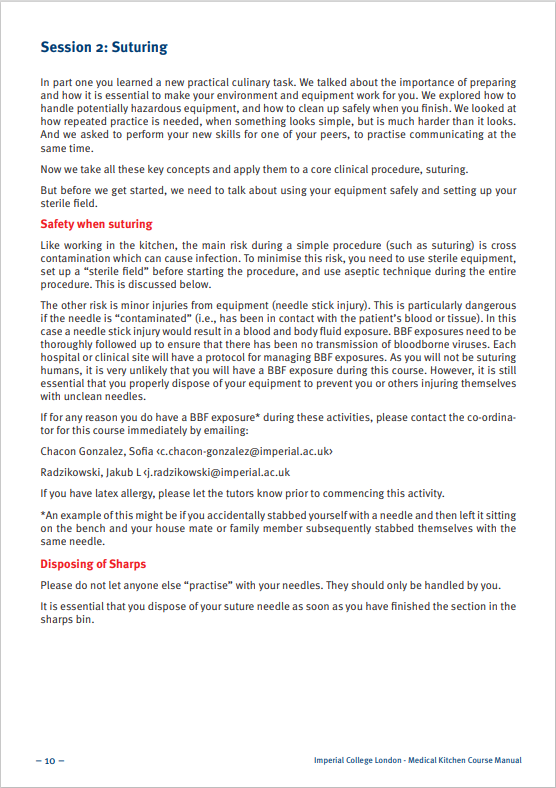

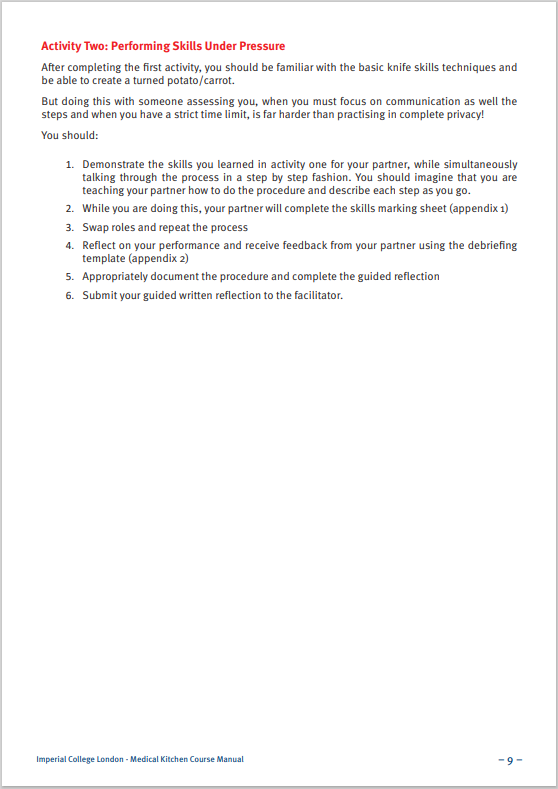


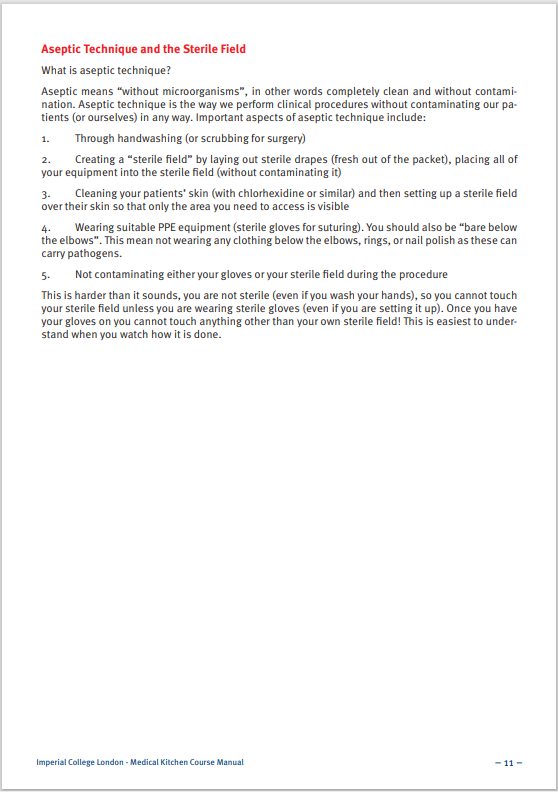

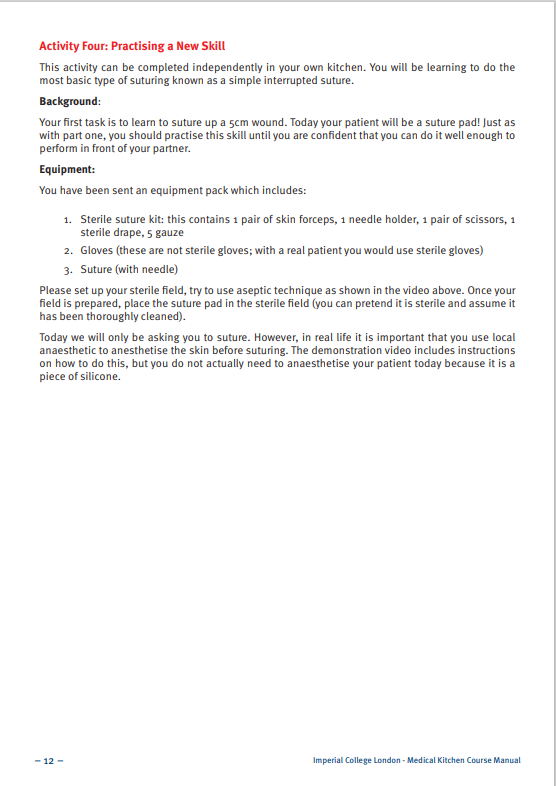


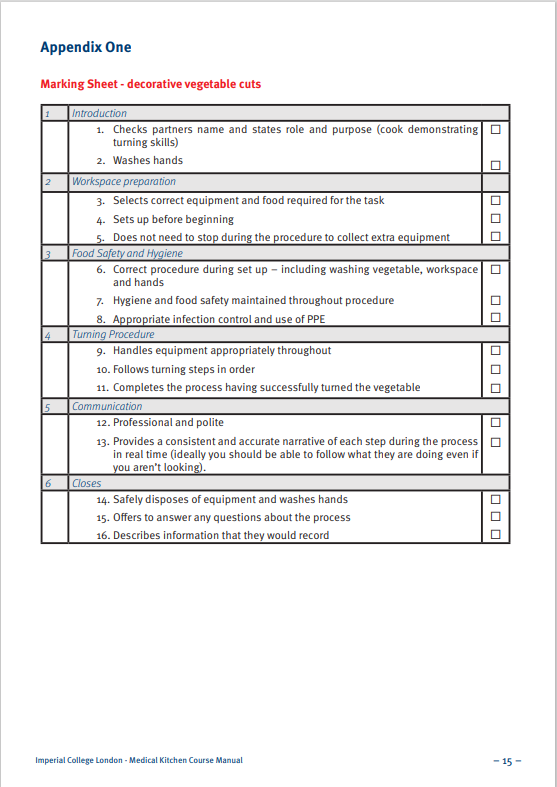

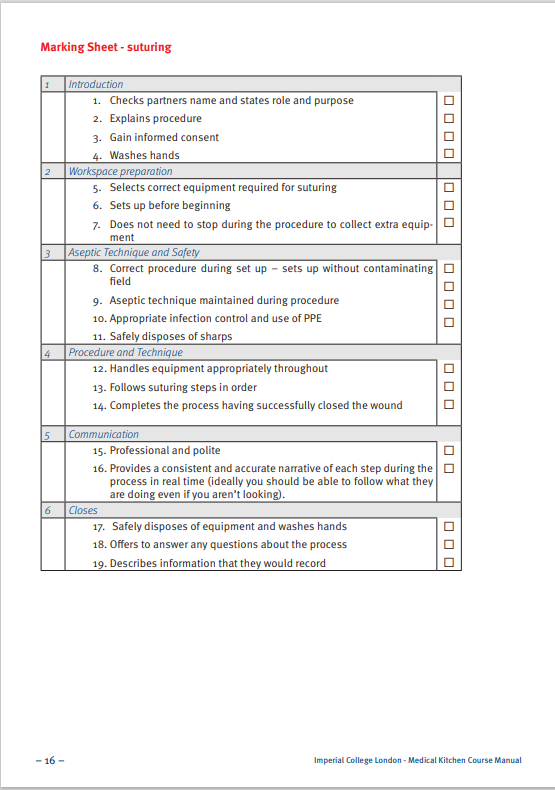


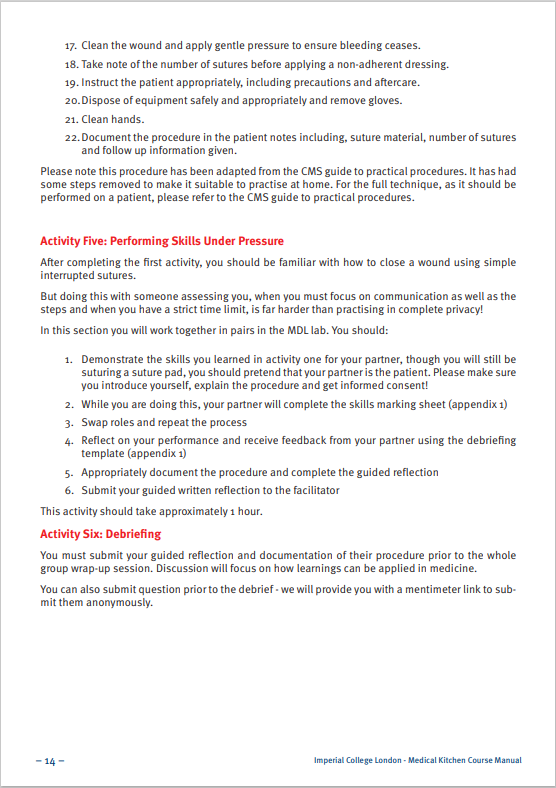

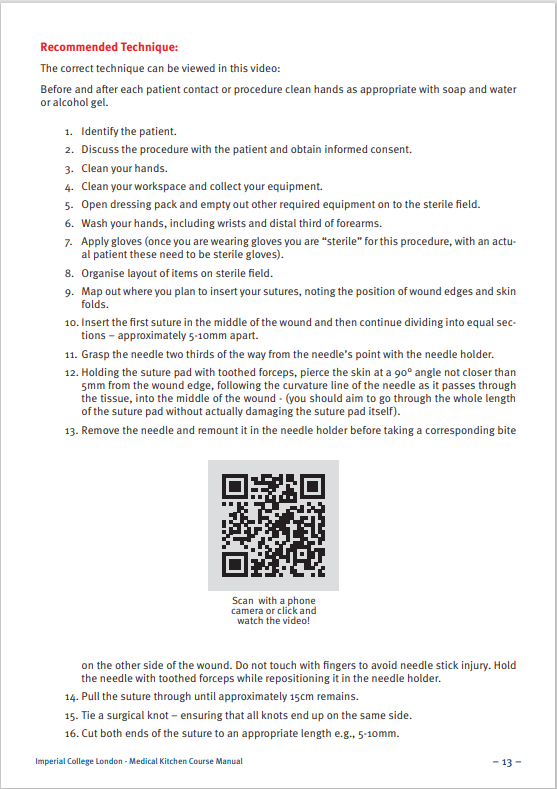


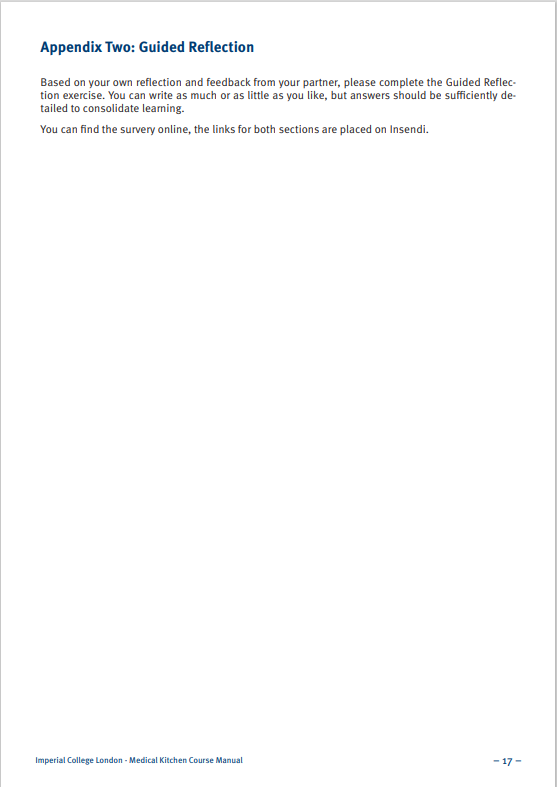


**Appendix 2: List of Questions for Focus Group Discussion**

**Key Questions of Focus Group**

1. Opening question: What is the first thing you recognise from the Medical Kitchen course?
2. Before attending, how did you hope the course would prepare you for your future practice?
3. Describe your most significant learning experience(s) in the course
   1. Which skill or component taught in the course have you found particularly important for your future practice?
   2. How has the course enhanced your understanding or appreciation of this aspect?
4. Can you share an example of a key insight or skill you gained from the Medical Kitchen that you hadn’t fully recognised before?
   1. How do you see this influencing your approach to self-directed learning and planning for your future professional practice?
5. How have the interactions with your peers and instructors during the course influenced your learning experience?
6. Strengths: What do you like most about the course?
    What current strengths should the course build upon?
7. Areas for improvement: What key improvement could be made to the course and why?

What would your remove from and add to the course?

1. What is the most important thing you would like to tell the course committee as they work to enhance the course?
2. Based on your experience, what advice would you give to future participants of the Medical Kitchen course?
3. Ending question: Is there anything that we should have talked about but didn’t?
